# Patient's dissatisfaction with the public and private laboratory services in conducting HIV related testing in Tanzania

**DOI:** 10.1186/1472-6963-8-167

**Published:** 2008-08-07

**Authors:** SG Mfinanga, A Kahwa, G Kimaro, A Kilale, S Kivuyo, M Senkoro, B Ngowi, R Mtandu, B Mutayoba, E Ngadaya, K Mashoto

**Affiliations:** 1 Muhimbili Medical Research Centre, National Institute for Medical Research (NIMR), Dar es es Salaam, Tanzania; 2 Centre for International Health (CIH), University of Bergen, Bergen, Norway; 3 Haydom Lutheran Hospital, Mbulu District, Manyara Region, Tanzania; 4 National Institute for Medical Research (NIMR), Headquarters, Dar es Salaam, Tanzania

## Abstract

**Background:**

Patient's satisfaction with both private and public laboratory services is important for the improvement of the health care delivery in any country.

**Methods:**

A cross-sectional survey was conducted in 24 randomly selected health facilities with laboratories that are conducting HIV related testing, in Mainland Tanzania. The study assessed patient's satisfaction with the laboratory services where by a total of 295 patients were interviewed.

**Results:**

Of data analyzed for a varying totals from 224 to 294 patients, the percentage of dissatisfaction with both public and private laboratory services, ranged from 4.3% to 34.8%, with most of variables being more than 15%. Patients who sought private laboratory services were less dissatisfied with the cleanness (3/72, 4.2%) and the privacy (10/72, 13.9%) than those sought public laboratory service for the same services of cleanness (41/222, 18.5%) and privacy (61/222, 27.5%), and proportional differences were statistically significant (X^2 ^= 8.7, p = 0.003 and X^2 ^= 5.5, p = 0.01, respectively). Patients with higher education were more likely to be dissatisfied with privacy (OR = 1.8, 95% CI: 1.1–3.1) and waiting time (OR = 2.5, 95% CI: 1.5 – 4.2) in both private and public facilities. Patients with secondary education were more likely to be dissatisfied with the waiting time (OR = 5.2; 95%CI: 2.2–12.2) and result notification (OR = 5.1 95%CI (2.2–12.2) than those with lower education.

**Conclusion:**

About 15.0% to 34.8% of patients were not satisfied with waiting time, privacy, results notification cleanness and timely instructions. Patients visited private facilities were less dissatisfied with cleanness and privacy of laboratory services than those visited public facilities. Patients with higher education were more likely to be dissatisfied with privacy and waiting time in both private and public facilities.

## Background

Scaling up of prevention, care and treatment for HIV/AIDS is important for the treatment and control of the diseases. This means that, the diagnostic services, Voluntary Counseling and Testing (VCT), Prevention of Mother to Child Transmission (PMTCT) services, and increasing Antiretroviral (ART) availability and use should also be expanded [1]. However, this effort should go hand in hand with improved quality of services to meet patient's needs. The needs of the patients should be taken into consideration in the design and implementation of laboratory services. Disregard for patients' feedback may cause constant disruption of testing because a patient has to come several times for the results and treatment [2]. Any laboratory should have a written policy for patient's satisfaction, and should periodically measure and evaluate patient's satisfaction. Most often, management of health facilities fail to integrate the results of satisfaction assessments into the continuous improvement and strategic planning processes. Satisfaction is one of the outcome measures for health care and thus monitoring patient satisfaction is an important and useful quality improvement tool and is required by most clinical laboratories. The main objective of this survey was to determine quality of laboratory services using patient's satisfaction criteria, in both public and private health facilities conducting HIV related laboratory testing.

## Methods

A cross-sectional baseline survey was conducted from February to March 2007 and a total of 24 laboratories participated in the survey. These laboratories were randomly selected from a list of all public and privately owned laboratories from each zone in Tanzania mainland using the simple random sampling method. Only facilities with laboratory services qualified to conduct HIV related testing and provision of ARV were eligible for the survey.

Interviewers, who were research assistants and graduates from medical schools, administered questionnaires using semi open ended questionnaires. Satisfaction status was measured using the dichotomy method and indifferent responses were not allowed. The research tools were pre-tested at two government hospital laboratories and one private hospital. After the pilot study the tools were modified to suite needs of the survey.

We had planned to interview a total of 288 patients, twelve patients from each visited laboratory on a first come basis. However, we interviewed a total of 295 patients. In some facilities research assistants interviewed more than 12 patients because some patients insisted that they want to give their perspectives and others came with their spouses for HIV testing at time where the total of 12 patients had already been reached. The research assistants accepted to allow a few more so as to increase social acceptability of the study in the respective facilities. The number of interviewees in each facility ranged from 12 to 14 patients.

Patients were interviewed regarding the satisfaction on safe phlebotomy experience with minimal discomfort or after-effect, clear communication between patients and phlebotomists and clear instructions for self-collected samples.

### Data analysis

Data management and analysis were carried out using Epi-Info 6.4 and SPSS 10.0 for Windows software. After data cleaning the total did not add up to 295 owing to missing values as shown in table 1. The same reason applies to variables that do not add up to expected totals in each subgroup of education levels as shown in table 2. Gender and age grouping showed no significant association with dissatisfaction with laboratory services in all satisfaction indicator variables. Private and public laboratories were used as a comparison groups for various satisfaction indicator variables as shown in result tables. Pearson Chi-squares were used to compare group differences of the categorical variables. Differences were considered statistically significant if p ≤ 0.05. Stratification and logistic regression analysis were carried out to assess and adjust for interaction and confounding effect of education on regions and turnout in either public or private laboratory. Adjusted odds ratios with 95% confidence intervals were reported where appropriate.

**Table 1:** Satisfaction and dissatisfaction with laboratory facility services

**Satisfaction with lab procedure**	**Type of Laboratory**		
	**Public**	**Private**	**ALL**

Phlebotomy	20/220(9.1)	4/71(5.6)	24/291(8.2)
Phlebotomist attitude	25/220(11.4)	5/71(7.0)	30/290(10.3)
Clear instructions	23/196 (11.7)	3/48(6.3)	26/224(10.7)
Timely Instructions	39/220 (17.7)	12/71(16.9)	51/291(17.5)
Cleanness	41/222(18.5)	3/72 (4.2)*	44/294(15.0)
Privacy	61/222 (27.5)	10/72(13.9)**	71/294(24.1)
			
Waiting time	81/221(36.7)	20/72(27.8)	101/294 (34.5)
Result Notification	78/218(35.8)	23/72(31.9)	101/290 (34.8)
Lab placement	10/212(4.7)	2/69(2.9)	12/281 (4.3)

* X^2 ^= 8.7, p = 0.003

**X^2 ^= 5.5, p = 0.01

**Table 2:** Dissatisfaction of laboratory procedure by educational level

**Risk Factors**	**Some education**	**Primary Education**	**Secondary Education**	**Higher Education**
Phlebotomy	9/50(18.0)	9/134(6.7)	3/54(5.6)	3/54(5.6)
Phlebotomist attitude	11/50(22.0)	11/134(8.2)	3/54(5.6)	5/55(9.3)
Clear instructions	7/55(15.6)	12/109(11.0)	4/46(8.7)	4/45(8.9)
Timely Instructions	8/50(16.0)	27/133(20.3)	9/55(16.7)	8/55(14.5)
Cleanness	6/50(12.0)	18/136(13.2)	9/54(16.7)	11/55(20.0)
Privacy	12/50(24.0)	25/136(18.4)	14/54(25.9)	20/55(36.4)
Waiting time	11/50(22.0)	39/136(28.7)	33/54(59.3)*	20/54(37.0)
Result Notification	1/46(2.2)	3/133(2.3)	6/51(11.8)*	2/52(3.8)

Laboratory placement	15/48(31.3)	43/135(31.9)	26/54(48.1)	18/54(33.3)

*p = 0.001 OR = 5.2 95% CI (2.2–12.2)

**p = 0.0001 OR = 5.1 95% CI (2.2–12.2)

### Ethical issues

Ethical clearance was obtained from National Institute for Medical Research Tanzania. Consent was sought from relevant administration of the hospital surveyed. Detailed information on the purpose of the survey and benefits were explicitly explained to each enrollee and that the participant is free to withdraw from the interview if they wished to do so. It was explained that if they decide to withdraw it will not have any effect on services provided to them. The informed consent was requested from each of personnel who was involved in the study

## Results

The mean age of study patients (295) from 10 regions of Tanzania was 35.6 (SD = 12.2) years. There was a statistical difference in the number of patients in the regions using public and private laboratories (X^2 ^= 79.1, df = 9, p value = 0.001). However, after controlling for education, a high proportion of patients from Arusha (OR = 4.8, 95% CI = 1.7–13.1) and Iringa (OR = 5.0, 95% CI = 2.4–10.5) were more likely to visit private laboratory services than did the patients in other regions. Majority of patients who had visited laboratory facilities had completed primary school education, married and were peasants. However, there was no statistical significance difference between private and public laboratory service users as regards to their demographic data. Most users of laboratory services visited the facilities for general check up (29.4%), CD4 counting (27%) and testing for HIV (17.3%).

Of data analyzed for a varying totals from 224 to 294 patients, the percentage of dissatisfaction with both public and private laboratory services, ranged from 4.3% to 34.8%, with most of variables being more than 15% (table 1).

Patients who sought private laboratory services were less dissatisfied with the cleanness (3/72, 4.2%) and the privacy (10/72, 13.9%) than those sought public laboratory service for the same services of cleanness (41/222, 18.5%) and privacy (61/222, 27.5%), and proportional differences were statistically significant (X^2 ^= 8.7, p = 0.003 and X^2 ^= 5.5, p = 0.01, respectively). Patients with higher education were more likely to be dissatisfied with privacy (OR = 1.8, 95% CI: 1.1–3.1) and waiting time (OR = 2.5, 95% CI: 1.5 – 4.2) in both private and public facilities. Patients with secondary education were more likely to be dissatisfied with the waiting time (OR = 5.2; 95%CI: 2.2–12.2) and result notification (OR = 5.1 95%CI (2.2–12.2) than those with lower education (table 2).

## Discussion

The study shows that a considerable higher percentage of the patients were dissatisfied with the laboratory services provided in the country including phlebotomy, phlebotomists' attitude, instructions, results notification, results communication, waiting time and privacy. This is contrary to results in developed countries where they have found high level of satisfaction with hospital services [3].

The main complaints were observed in waiting time, results notification, privacy and timely instruction. Additionally, level of privacy in the consultation room was described as unsatisfactory by 24.1% of patients, which compares with a study done in other country in Africa like Egypt [2]. In our study like in other studies done in Egypt and USA found no association between overall patient's dissatisfaction with age and gender [2,4]. However, we found an association between dissatisfaction and level of education and type of facility of either being public or private. In USA it has been shown that improving laboratory information system, (especially on turn around time), repeatedly patient satisfaction surveys, and continuous monitoring of providers of laboratory services can improve quality laboratory services [1,4,5]. This can also be done in our country to improve quality of laboratory services in those aspects which patients were not satisfied.

Substantial proportions of the patients were not satisfied with the waiting time and result notification. Patients wanted to receive their results in timely manner, shortly after the physician or provider receives the results. Baldwin and his colleagues (2005) uncovered that patient's privacy, assured confidentiality of test results and diagnosis is of importance to the patient. Thus, privacy, responsive and interactive feedback, convenience, and timeliness with detailed information may be critical for patient satisfaction and for improving patient safety [6]. The competitive forces in today's health care environment require medical practices to address issues related to patient's satisfaction [7]. In this survey we have found that users of private laboratories were more likely to be satisfied with cleanness and privacy than the public laboratory users. Most of buildings in public health facilities are very old and difficult to clean. Rarely the medical personnel in public health facilities spare enough time to talk with patients in privacy, perhaps due to long time shortage of human resources in this sector. However highly educated patients were more likely to be dissatisfied with privacy and waiting time. Gone are the days when most patients tolerated impersonal service from their health-care providers in general. Every day, patient's satisfaction becomes more critical to a health-care provider's success and survival. We therefore recommend interventions to improve quality of laboratory services, especially, in public health services.

The study involved all laboratories that conduct HIV related testing and therefore caution should be taken when inferring to other laboratories in the country. We also admit the fact that, we slightly interviewed more patients than expected by about 2.4%. This is a small increase and is not likely to influence our findings in either way because the percentage of missing value is low to have noticeable effect on our findings. Also for practical reasons, patients were selected on a first come basis and the dichotomy method was used instead of Likerts method, which could have probably affected the findings in either way toward satisfaction or dissatisfaction. However, since this study involved several facilities in different regions, including rural and urban settings, it is less likely to have unforeseen biasing factor that could have constantly operated in all visited facilities. Therefore, the effect of this phenomenon could have less impact in the findings.

## Conclusion

About 15.0% to 34.8% of patients were not satisfied with waiting time, privacy, results notification cleanness and timely instructions. Patients visited private facilities were less dissatisfied with cleanness and privacy of laboratory services than those visited public facilities. Patients with higher education were more likely to be dissatisfied with privacy and waiting time in both private and public facilities.

## Competing interests

The authors declare that they have no competing interests.

## Authors' contributions

SGM: Provided contribution on the study design, performed statistical analysis, drafted the manuscript and responded to the reviewer's comments. He also gave final approval of the version to be published. AK: Participated in the design of the study, coordinating, drafting the manuscript, and responding to the reviewer's comments. GK: Participated in the study design, data collection and revising the manuscript. KA: Involved in drafting the manuscript and revising it critically. MS: Provided substantial contributions to conception and design, acquisition of data, analysis and interpretation of data. SK: Participated in data collection, analysis and final approval of the version to be published. BN: Participated in the study design and data collection. RM: Participated in data collection, drafting the manuscript and revising it critically for important intellectual content. BM: Have made substantial contributions to conception and design of the study, acquisition of data, analysis and interpretation. EN: Participated in the design of the study, coordinating, drafting the manuscript, and responding to the reviewer's comments. KM: Involved in analysis drafting the manuscript and revising it critically for important intellectual content

## Pre-publication history

The pre-publication history for this paper can be accessed here:


